# Editorial: Pests and diseases monitoring and forecasting algorithms, technologies, and applications

**DOI:** 10.3389/fpls.2024.1518814

**Published:** 2024-12-04

**Authors:** Yingying Dong, Wenjiang Huang, Kejian Lin, Liangxiu Han, Giovanni Laneve, Jingcheng Zhang

**Affiliations:** ^1^ Aerospace Information Research Institute, Chinese Academy of Sciences (CAS), Beijing, China; ^2^ Institute of Grassland Research, Chinese Academy of Agricultural Sciences (CAAS), Hohhot, China; ^3^ Department of Computing, and Mathematics, Manchester Metropolitan University, Manchester, United Kingdom; ^4^ School of Aerospace Engineering, Sapienza University of Rome, Rome, Italy; ^5^ College of Artificial Intelligence, Hangzhou Dianzi University, Hangzhou, China

**Keywords:** pest and disease monitoring, AI and machine learning, UAV technology, multispectral and hyperspectral data, precision agriculture

In the face of growing challenges in agriculture due to pests and diseases, the need for advanced monitoring and forecasting techniques has become increasingly critical. Climate change, global trade, and the adaptation of pests to traditional control methods have further complicated this landscape. This Research Topic offers a collection of studies highlighting the latest advancements in pest and disease monitoring, focusing on the development and application of innovative algorithms, technologies, and practical solutions to mitigate the impact of these threats on agriculture.

Recent advances in deep learning, such as fast Fourier Convolutional Networks, have shown promise in distinguishing between similar symptoms like wheat yellow rust and nitrogen deficiency using Sentinel-2 time series data (Shi et al.). These techniques underscore the power of modern AI to refine diagnostic accuracy, which is crucial for early intervention and targeted management. Similarly, the spatial ensemble model has been employed to assess the potential risk zones of Pierce’s disease across Europe, integrating multiple data sources to offer more reliable predictions for pest management (Yoon et al.).

Within controlled environments, greenhouse-based pest monitoring has seen significant improvements due to the implementation of deep learning and machine vision (Zhang et al.). Automatic identification systems are now capable of real-time recognition of pests, thanks to the deployment of sophisticated neural networks. The integration of UAV technology with deep learning also extends pest monitoring capabilities to broader agricultural landscapes. For instance, studies on Brandt’s vole detection and counting via UAV-based systems exemplify the ability to efficiently monitor field conditions (Wu et al.), while multispectral imaging from UAVs provides detailed nutritional assessments, such as potassium levels in potato plants (Ma et al.).

Machine learning architectures have also been developed to handle complex diagnostic tasks in challenging environments (Liu et al.). Techniques like the multi-scale double-branch GAN-ResNet for rice pest identification demonstrate the application of advanced algorithms in complex scenarios, including those with variable backgrounds (Hu et al.). Other lightweight deep learning models, such as MS-Net, are designed to be both accurate and efficient, focusing on optimizing computational resources without compromising precision (Quan et al.).

The fusion of multispectral and hyperspectral data has shown great potential in early disease detection across different crop types and ecosystems. The MSGF-GLP method, for example, utilizes visible and hyperspectral data to identify stressed vegetation, enhancing early detection capabilities (Zhou et al.). These approaches highlight the increasing role of spectral data in disease management, offering more nuanced insights into plant health (Huang et al.).

Field-based applications of these technologies have also made considerable strides. Studies on the penetration of fog droplets in fruit tree canopies (Sun et al.) reveal the multifactorial elements affecting pesticide delivery efficiency. These findings are crucial for improving precision agriculture, allowing targeted interventions that minimize pesticide use while maximizing coverage. Lightweight models like the enhanced CNN (Dai et al.) for pepper leaf disease recognition showcase how AI can be applied to specific crops, even in complex agricultural settings. Similarly, research on weed identification in soybean fields using lightweight segmentation models such as DCSAnet demonstrates (Yu et al.) the application of optimized AI architectures in practical field conditions.

The rise of mobile applications powered by AI, such as GranoScan (Dainelli et al.) for in-field wheat threat identification, reflects the growing trend of democratizing technology for farmers. These tools provide accessible and accurate diagnostic capabilities, empowering agricultural stakeholders with real-time data. Likewise, the development of UAV spraying systems (Liu et al.) that account for pest activity patterns, such as thrips during the cotton flowering period, illustrates the synergy between automated technologies and pest behavior research. Additionally, a risk-based regionalization approach has been proposed for the area-wide management of HLB vectors in the Mediterranean Basin (Galvan et al.), offering a strategic perspective to mitigate the spread of disease.

The Research Topic also addresses challenges associated with AI applications in pest monitoring. Issues such as complex environments, small object detection, and the variability of natural conditions continue to test the limits of current technologies. Innovations like the Skip DETR model (Liu et al.), which integrates skip connections for small object detection, and the adaptive filtering fusion method for pest recognition, indicate ongoing efforts to overcome these obstacles (Chen et al.).

In summary, this Research Topic offers a comprehensive overview of current innovations in pest and disease monitoring. The articles included emphasize the growing role of AI, machine learning, and advanced imaging technologies in modern agriculture. Together, these studies not only demonstrate the effectiveness of cutting-edge solutions but also underline the importance of continued collaboration across disciplines to address the evolving challenges in pest and disease management.

